# Predictor for supraphysiologic serum estradiol elevation on hCG triggering day of controlled ovarian stimulation using letrozole and gonadotropins in women with estrogen-dependent cancers

**DOI:** 10.1371/journal.pone.0240870

**Published:** 2020-10-21

**Authors:** Sung Woo Kim, Soo Jin Han, Ji Yeon Han, Hoon Kim, Seung-Yup Ku, Chang Suk Suh

**Affiliations:** 1 Department of Obstetrics and Gynecology, Seoul National University Hospital, Seoul, South Korea; 2 Institute of Reproductive Medicine and Population, Medical Research Center, Seoul National University, Seoul, South Korea; Michigan State University, UNITED STATES

## Abstract

The objective of this study was to evaluate predicting factors for supraphysiologic serum estradiol elevation during controlled ovarian stimulation (COS) with administration of letrozole and gonadotropins in patients with estrogen-dependent cancer. Use of aromatase inhibitors is recommended to prevent the potential effects of elevated serum estradiol levels and recurrence of tumor in patients with estrogen-dependent cancers during COS. Although previous studies reported that letrozole have shown an effective lowering of peak estrogen levels, a part of patients shows supraphysiologic levels of estrogen associated with ovarian stimulation despite the administration of letrozole. From January 2009 to December 2019, patients with estrogen-dependent cancer who underwent COS with antagonist protocol using a letrozole (5 mg/ day) to keep estrogen levels low were included in this study. Early monitoring serum estradiol was measured in all patients on the 4-6th day of stimulation. Subjects were classified into two groups according to the serum estradiol level on hCG triggering day, physiologic estradiol group (≤400 pg/mL) and supraphysiologic estradiol group (>400 pg/mL). A total of 96 COS cycles were retrospectively analyzed. Supraphysiologic level of serum estradiol was found in 21.9% of the patients. Mean age, AMH, duration of stimulation, total dose of gonadotropins administered were not different between the two groups. However, early monitoring serum estradiol level was significantly higher in the supraphysiologic estradiol group (67.1±47.9 vs. 115.6±78.1, p = 0.001) and was associated with the occurrence of supraphysiologic elevation of serum estradiol on hCG triggering day. Patients with early monitoring serum estradiol ≥84.5 pg/mL had an odds ratio of 5.376 [95% CI, 1.613–17.913] for supraphysiologic elevation of serum estradiol compared to those with early monitoring serum estradiol below 84.5 pg/mL. In conclusion, early monitoring serum estradiol is an independent predicting factor for supraphysiologic level of serum estradiol on hCG triggering day in the COS cycles using letrozole and gonadotropins.

## Introduction

The population of estrogen-dependent cancers which includes breast cancer and endometrial cancer is growing recently and a considerable number of patients are diagnosed at their reproductive age [1]. Breast cancer is the most commonly diagnosed type of cancer in women accounting for 29% of all female cancer and uterine corpus cancer is the fourth among female cancer accounting for 6% in USA [2, 3]. Invasive breast cancer affects 0.5% of women under 40 years of age [3]. Increasing cancer survival rates as a consequence of improved surgical treatments and chemotherapies have made both clinicians and patients more care about quality of life in future such as family planning and childbearing [4, 5].

Potential options for fertility preservation (FP) in women with cancer includes embryo cryopreservation, oocyte cryopreservation, ovarian tissue cryopreservation and maturation of oocytes in vitro [6]. Among the fertility preservation options, embryo cryopreservation is the most established method which provides 25–35% possibility of pregnancy [7]. As the pregnancy rate and perinatal outcome are improved through the introduction of vitrification, the number of oocyte cryopreservation for the purpose of fertility preservation before chemotherapy is increasing in unmarried women [8].

During the conventional controlled ovarian stimulation (COS) during in vitro fertilization (IVF), serum estradiol concentration may increase to 10 to 15-fold higher level than physiologic range since the growth of multiple follicles are achieved by gonadotropins [9, 10]. Supraphysiologic serum E2 levels (≥400 pg/mL) may promote the tumor growth not only in the estrogen receptor (ER) positive breast cancer but also in the ER negative breast cancer [11]. To prevent the elevation of serum estrogen levels and following potential risks in estrogen-dependent cancer patients, alternative COS methods have been studied to reduce the adverse effects on tumors during COS [12–15].

The peak serum estradiol level was lower when administrating of letrozole, a type of aromatase inhibitor, simultaneously in conventional COS protocol compared to the tamoxifen or anastrozole [16, 17]. Therefore the protocol of administrating letrozole (5 mg/day) orally from two days before starting gonadotropin during COS is commonly used [18]. Whereas the combined use of letrozole with gonadotropins provided reduction in total gonadotropin requirement as well as significantly lower peak estradiol level, compared to the conventional IVF, the number of oocytes retrieved and the maturity of oocytes were comparable [19]. In addition, frozen-thawed embryo transfer in women with breast cancer who underwent embryo cryopreservation after COS with letrozole and gonadotropins for the purpose of FP resulted a similar outcome in live birth rate per embryo transfer compared to the infertile women of a similar age [18, 20].

However, peak serum estradiol level rises above the physiologic level (>400 pg/mL) in some patients even though 5 mg of letrozole is administered during COS. There is a concern that supraphysiologic estrogen level may adversely affect the growth of tumor in patients scheduled to undergo neoadjuvant chemotherapy, and the recurrence of tumor in patients supposed to start adjuvant chemotherapy. Nevertheless, to date, predictors for supraphysiologic serum estradiol elevation in COS with letrozole and gonadotropins have not been reported.

The identification of predictor is beneficial for preventing supraphysiologic serum estradiol elevation and clinician can proceed COS safely by reducing the potential negative effect on estrogen-dependent cancers. The objective of this study was to investigate the factors predicting supraphysiologic serum estradiol elevation on hCG triggering day of COS using letrozole and gonadotropins in women with estrogen-dependent cancers.

## Materials and methods

### Study design and subjects

This is a retrospective cohort study including patients with estrogen-dependent cancers who underwent COS for FP or IVF in a single tertiary center (Seoul National University Hospital, Republic of Korea) from January 2009 to December 2019. Both conventional-start cycles and random-start cycles were included in the study. Early monitoring serum E2 and peak serum E2 were measured on the 4−6th day of stimulation and on hCG triggering day respectively in all patients. Serum estradiol level reaches peak concentration as high as 400 pg/mL at the time of pre-ovulation in natural menstrual cycle [21–23]. Therefore, the study population was divided into two groups according to peak serum E2 level; patients with peak serum E2 level ≤400 pg/mL were included in physiologic E2 group, whereas those with peak serum E2 level >400 pg/mL were assigned to supra-physiologic E2 group. The institutional review boards of Seoul National University Hospital approved this study (No. H-1912-127-1091), and informed consent was acquired verbally.

### Ovarian stimulation and letrozole protocol

Ovarian stimulation began with a dose of 150–450 IU/day on the second or third day of the menstrual cycle in conventional-start cycles or immediately started after referral in random-start cycles without awaiting menstruation in the patients required emergent fertility preservation. All COS cycles were performed using GnRH antagonist protocol with a fixed dose of letrozole (5 mg/day) as an adjuvant to exogenous gonadotropins to keep estrogen levels low during ovarian stimulation. Administration of letrozole (Femara^®^, Novartis) was started two days before or on the starting day of ovarian stimulation and was continued until the hCG triggering day. Dose of recombinant FSH (Gonal-F^®^, Merck Serono) was adjusted according to the patient’s age, anti-Mullerian hormone (AMH), and body mass index (BMI). GnRH antagonist cetrorelix 0.25 mg (Cetrotide^®^, Merck Serono) was administered from when the leading follicle reached a mean diameter of 14 mm. As soon as at least 2 follicles with a mean diameter of ≥18−20 mm was observed, final oocyte maturation was achieved with either a recombinant hCG (Ovidrel^®^, 250 μg, Merck Serono) or 10,000 IU of highly purified urinary hCG (IVF-C^®^, LG Chem). Oocytes were retrieved by transvaginal aspiration 36 hours after hCG triggering.

### Outcome measures

Serum estradiol levels were determined using Chemiluminescent Microparticle Immuno Assay (CMIA, Architect, Abbott). Age, BMI, the number of follicles ≥10 mm, ≥12 mm and ≥14 mm on the 4−6th day of stimulation, duration of stimulation, total dose of recombinant FSH administered, total dose of letrozole administered, the number of oocytes retrieved and the number of mature oocytes retrieved were evaluated.

### Statistical analysis

COS parameters are presented as mean ± standard deviation (SD) and compared between cycles with physiologic peak E2 and supraphysiologic peak E2 using the Student’s t test. Univariate analysis was performed to select predictor candidates for supraphysiologic serum E2 elevation. Significant variables in the univariate analysis were included in the multivariate analysis to determine the independent predictor for supraphysiologic serum E2 elevation. In addition, receiver operating characteristic (ROC) analysis was used to determine the cut-off level of early monitoring serum E2 predicting the occurrence of supraphysiologic level of serum E2. Logistic regression performed to calculate odds ratios (ORs) and corresponding 95% confidence intervals (CIs) for the association between the cut-off level of early monitoring serum E2 and the risk of supraphysiologic serum E2 elevation. *P* value of <0.05 was considered statistically significant. All statistical analyses were performed using SPSS software version 25.0 (SPSS Inc., Chicago, IL, USA).

## Results

### Patients

During the study period, a total of 96 antagonist COS cycles were performed in hormone-dependent cancer patients. The diagnosis of hormone-dependent cancers included in this study was breast cancer (n = 85), endometrial cancer (n = 11). In all cycles, a fixed dose of letrozole (5 mg/day) was administered simultaneously during ovarian stimulation to keep low serum estrogen level.

**Fig 1:**
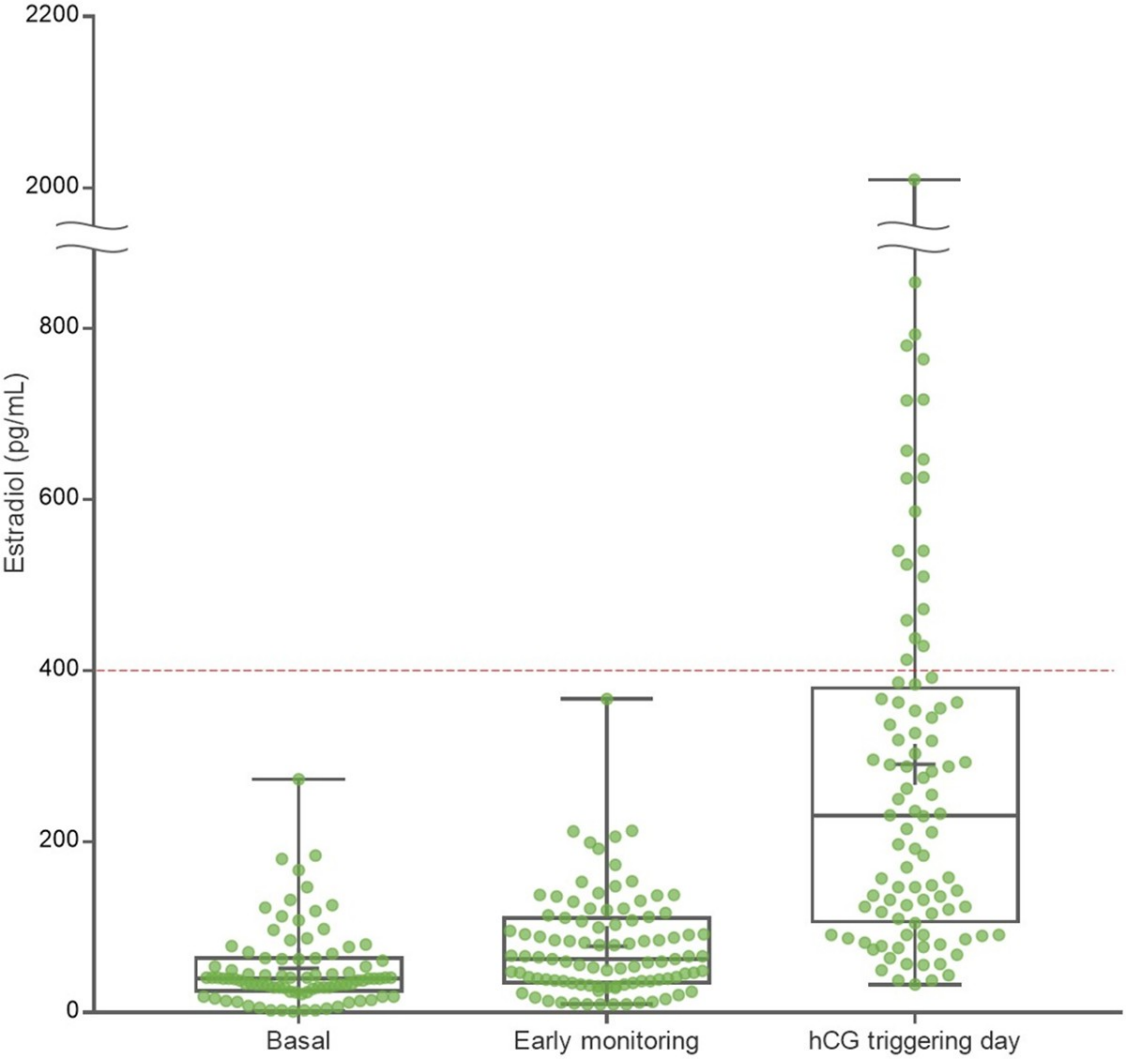
Estradiol level during controlled ovarian stimulation with letrozole and gonadotropins.

Baseline characteristics of all subjects of this study are presented in Table 1. Mean age was 34.7(±4.9) years, mean anti-Müllerian hormone (AMH) level was 4.0(±4.1) ng/mL, mean early monitoring serum E2 level was 77.7(±59.0) pg/mL and mean peak E2 level on hCG triggering day was 290.3(±271.3) pg/mL which was within the physiologic level (Fig 1).

**Table 1:** Baseline characteristics of study subjects.

	Number (%)
Cancer types	
Breast cancer	85 (88.5)
Endometrial cancer	11 (11.5)
Starting phase	
Conventional start	41 (42.7)
Random start	55 (57.3)
Purpose of ovarian stimulation	
Fertility preservation	65 (67.7)
Infertility treatment	31 (32.3)
	Mean±standard deviation
Age (yr)	34.7±4.9
BMI (kg/m^2^)	22.9±3.9
AMH (ng/mL)	4.0±4.1
Basal LH (mIU/mL)	6.8±16.8
Basal FSH (mIU/mL)	5.5±3.2
Basal E2 (pg/mL)	52.0±46.5
Early monitoring serum E2 (pg/mL)	77.7±59.0
Early monitoring No. of follicles ≥10 mm	2.0±2.2
Early monitoring No. of follicles ≥12 mm	0.8±1.3
Early monitoring No. of follicles ≥14 mm	0.3±0.7
Duration of ovarian stimulation (days)	8.4±2.2
Total dose of gonadotropins (IU)	2568.0±1307.8
Starting dose of gonadotropins (IU)	287.9±108.9
Total dose of letrozole (mg)	47.1±10.8
Duration of letrozole administered (days)	9.4±2.2
E2 on hCG triggering day (pg/mL)	290.3±271.3
P4 on hCG triggering day (ng/mL)	1.4±1.8
No. of oocytes retrieved	9.0±6.5
MII rate	0.55±0.30

BMI, body mass index; AMH, anti-Mullerian hormone; LH, luteinizing hormone; FSH, follicle-stimulating hormone; E2, estradiol; P4, progesterone

### Predictors of estradiol elevation

Supraphysiologic level of serum E2 was found in 21.9% of all cycles. Mean age, AMH, basal serum LH/ FSH/ E2, total dose and duration of letrozole administered, duration of stimulation, total dose of gonadotropins administered were not different between the physiologic E2 group (peak E2 ≤400 pg/mL) and supraphysiologic E2 group (peak E2 >400 pg/mL) (Table 2). However, BMI, early monitoring serum E2 level and number of follicles ≥10mm were significantly different between the two groups (23.2±4.2 vs. 21.8±2.0, *p* = 0.045; 67.1±47.9 vs. 115.6±78.1, *p* = 0.001; 1.7±2.0 vs. 3.1±2.8, *p* = 0.009, respectively).

**Table 2:** Comparison of variables between cycles with and without supraphysiologic level of serum E2 (>400 pg/ml) on the day of hCG administration.

	Physiologic E2 (n = 75, 78.1%)	Supra-physiologic E2 (n = 21, 21.9%)	*p*
Age (yr)	34.7±5.0	34.4±4.9	0.782
BMI (kg/m^2^)	23.2±4.2	21.8±2.0	0.045*
AMH (ng/mL)	3.9±4.3	4.2±3.1	0.736
Basal LH (mIU/mL)	7.4±19.0	4.7±3.3	0.529
Basal FSH (mIU/mL)	5.6±3.3	5.0±3.1	0.502
Basal E2 (pg/mL)	47.6±44.5	66.2±50.9	0.108
Early monitoring serum E2 (pg/mL)	67.1±47.9	115.6±78.1	0.001*
Early monitoring No. of follicles ≥10 mm	1.7±2.0	3.1±2.8	0.009*
Early monitoring No. of follicles ≥12 mm	0.7±1.2	1.2±1.9	0.107
Early monitoring No. of follicles ≥14 mm	0.3±0.7	0.4±0.8	0.309
Duration of ovarian stimulation (days)	8.5±2.4	8.3±1.6	0.658
Total dose of gonadotropins (IU)	2655.5±1354.7	2255.4±1096.7	0.217
Starting dose of gonadotropins (IU)	293.0±109.2	269.6±108.4	0.388
Total dose of letrozole (mg)	47.1±11.6	47.1±7.3	0.977
Duration of letrozole administered (days)	9.4±2.3	9.4±1.5	0.977
E2 on hCG triggering day (pg/mL)	183.6±108.0	671.4±333.5	0.000
P4 on hCG triggering day (ng/mL)	1.2±1.7	1.9±2.0	0.116
No. of oocytes retrieved	8.0±6.0	12.4±7.4	0.007*
MII rate	0.56±0.30	0.53±0.31	0.685
Cancer types			0.062
Breast cancer	64 (85.3)	21 (100)	
Endometrial cancer	11 (14.7)	0 (0)	
Starting phase			0.311
Conventional start	30 (40.0)	11 (52.4)	
Random start	45 (60.0)	10 (47.6)	
Purpose of ovarian stimulation			0.680
Fertility preservation	50 (66.7)	15 (71.4)	
Infertility treatment	25 (33.3)	6 (28.6)	

Data are presented as mean±SD or number (%)

BMI, body mass index; AMH, anti-Mullerian hormone; LH, luteinizing hormone; FSH, follicle-stimulating hormone; E2, estradiol; P4, progesterone

**Fig 2:**
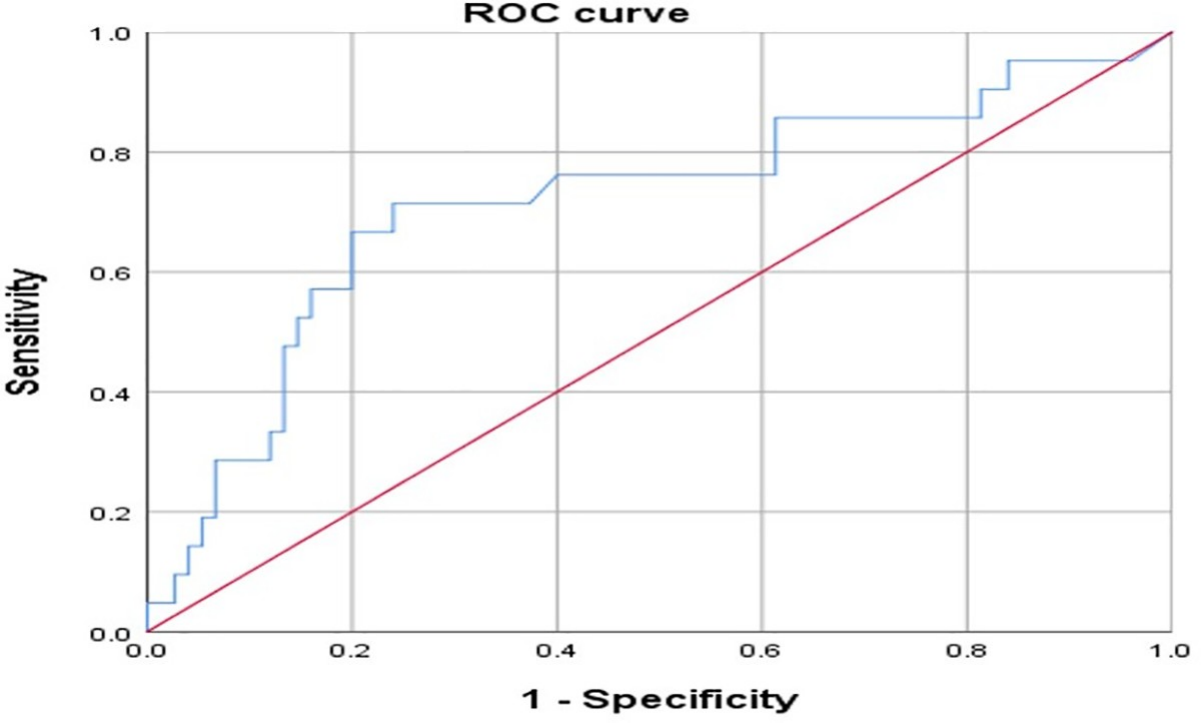
ROC curve of early monitoring serum E2 for predicting supraphysiologic E2 elevation (>400 pg/ml) on the day of hCG administration.

**Fig 3:**
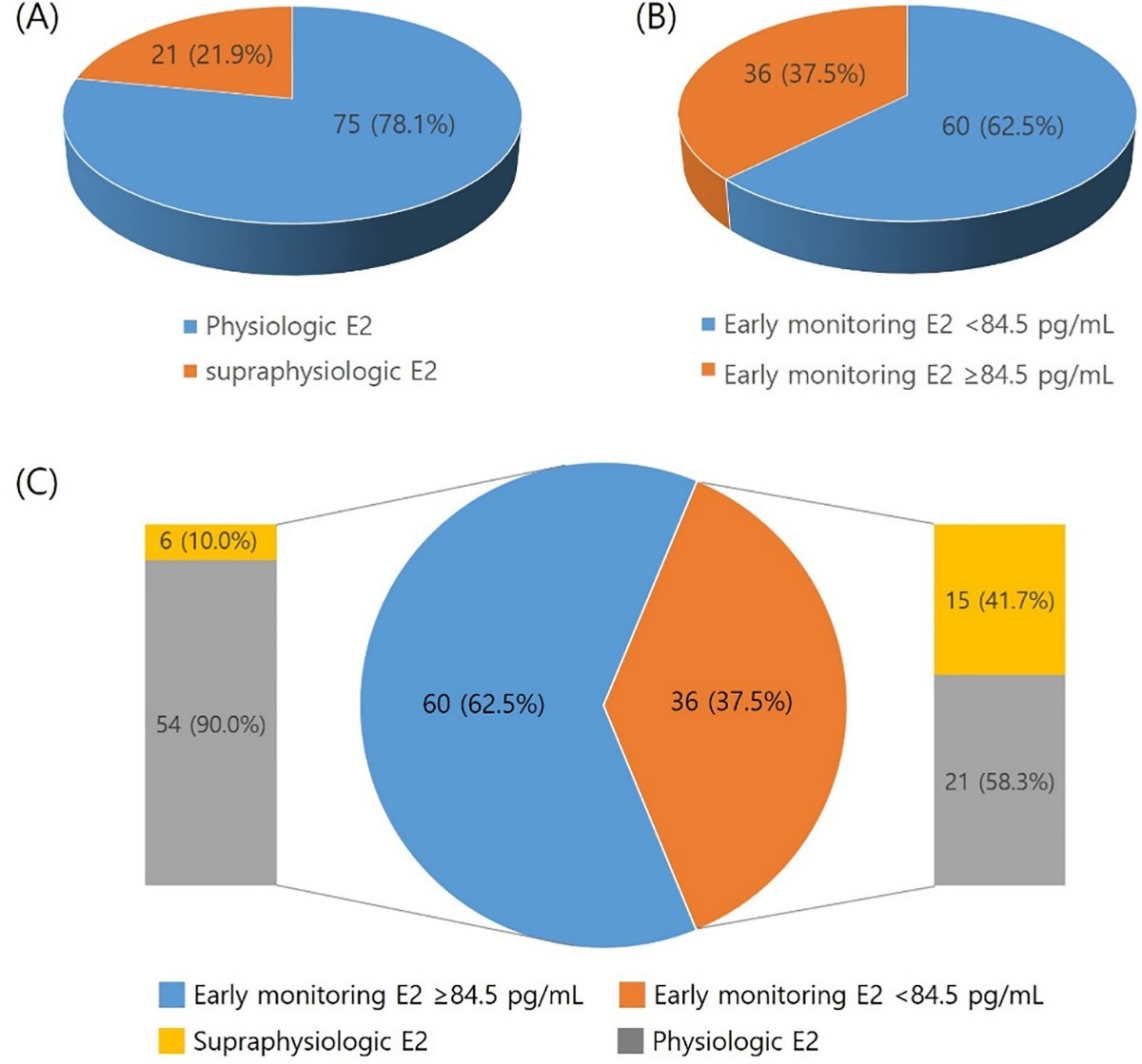
Proportion of early monitoring serum E2 (≥84.5 pg/mL) and SP E2 (>400 pg/ml). (A) Proportion of cycles with supraphysiologic serum E2 level on the hCG triggering day. (B) Early monitoring serum E2 according to the cut-off level (≥84.5 pg/mL). (C) Proportions of supraphysiologic serum E2 according to the cut-off level of early monitoring serum E2.

Univariate logistic regression analysis showed that early monitoring serum E2 level was significantly associated with the occurrence of supraphysiologic elevation of serum E2 (Table 3). Early monitoring serum E2 is associated with the occurrence of supraphysiologic elevation of serum E2 on hCG triggering day after controlling for BMI, early monitoring number of follicles ≥ 10 mm and starting phase of protocol (adjusted odds ratio [aOR] = 1.010; 95% confidence interval [CI], 1.000–1.020) (Table 4). Fig 2 is the receiver operating characteristic (ROC) curve of early monitoring serum E2 for predicting occurrence of supraphysiologic estrogen levels. The mean area under the ROC curve (AUC) is 0.716 (p = 0.003 compared with 0.5). The best cut-off for early monitoring serum E2 to predict the occurrence of supraphysiologic estrogen levels is 84.5 pg/mL and at that point, the sensitivity was 71.4% and specificity was 72.0%. Patients with early monitoring serum E2 ≥84.5 pg/mL had an odds ratio of 5.372 [95% CI, 1.597–18.072] for supraphysiologic elevation of serum E2 compared to who with early monitoring serum E2 below 84.5 pg/mL (Table 5) (Fig 3).

**Table 3:** Univariable logistic regression analyses of the association between baseline/cycle variables and the occurrence of supraphysiologic E2 (>400 pg/ml) on the day of hCG administration.

	B	*P*	OR	95% CI
Age (yr)	-0.014	0.780	0.986	0.893–1.088
BMI (kg/m^2^)	-0.105	0.169	0.900	0.775–1.045
AMH (ng/mL)	0.020	0.733	1.020	0.909–1.145
Basal LH (mIU/mL)	-0.024	0.583	0.977	0.898–1.063
Basal FSH (mIU/mL)	-0.056	0.498	0.945	0.804–1.112
Basal E2 (pg/mL)	0.008	0.122	1.008	0.998–1.018
Early monitoring serum E2 (pg/mL)	0.013	0.004*	1.014	1.004–1.023
Early monitoring No. of follicles ≥10 mm	0.259	0.015*	1.296	1.051–1.598
Early monitoring No. of follicles ≥12 mm	0.253	0.127	1.288	0.931–1.781
Early monitoring No. of follicles ≥14 mm	0.318	0.317	1.374	0.738–2.558
Duration of ovarian stimulation (days)	-0.041	0.721	0.960	0.766–1.203
Total dose of gonadotropins (IU)	0.000	0.219	1.000	0.999–1.000
Starting dose of gonadotropins (IU)	-0.002	0.385	0.998	0.993–1.003
Total dose of letrozole (mg)	0.001	0.977	1.001	0.957–1.047
Duration of letrozole administered (days)	0.003	0.977	1.003	0.801–1.257
Cancer type			N/A	
Starting phase				
Conventional start			1.000	
Random start	-0.501	0.313	0.606	0.229–1.604
Purpose of ovarian stimulation				
Fertility preservation			1.000	
Infertility treatment	-0.223	0.680	0.800	0.277–2.313

BMI, body mass index; AMH, anti-Mullerian hormone; LH, luteinizing hormone; FSH, follicle-stimulating hormone; E2, estradiol; N/A, not applicable

**Table 4:** Multivariable logistic regression analyses of the association between baseline/cycle variables and the occurrence of supraphysiologic E2 (>400 pg/ml) on the day of hCG administration.

	B	*P*	OR	95% CI
BMI (kg/m^2^)	-0.081	0.348	0.923	0.780–1.092
Early monitoring serum E2 (pg/mL)	0.010	0.043*	1.010	1.000–1.020
Early monitoring No. of follicles ≥10 mm	0.124	0.304	1.132	0.893–1.435
Starting phase of protocol	-0.040	0.945	0.961	0.310–2.976

BMI, body mass index; E2, estradiol

**Table 5:** Supraphysiologic E2 according to cut-off value of early monitoring phase E2 (≥84.5 pg/mL).

	B	*P*	OR	95% CI
BMI (kg/m^2^)	-0.107	0.230	0.898	0.754–1.070
Early monitoring No. of follicles ≥10mm	0.090	0.470	1.095	0.857–1.399
E2 (≥84.5 pg/ml)	1.681	0.007*	5.372	1.597–18.072
Starting phase of protocol	-0.006	0.992	0.994	0.311–3.182

BMI, body mass index; E2, estradiol

## Discussion

This is the first study to elucidate the factors predicting supraphysiologic serum E2 elevation on hCG triggering day in estrogen-dependent cancer patients undergoing COS using letrozole and gonadotropins. Early monitoring serum E2 levels measured on the early period of stimulation were found to be an independent predictor for supraphysiologic serum E2 elevation, and the present study determined the best cut-off for early monitoring serum E2 to predict the occurrence of supraphysiologic serum E2 elevation. To the best of our knowledge, so far, few studies reported that serum E2 level measured in the early stimulation period is a factor that can independently predict E2 elevation above physiological concentration on hCG triggering day. Patients with early monitoring serum E2 ≥84.5 pg/mL were at high-risk for supraphysiologic serum E2 level on hCG triggering day. Strategies for predicting and preventing supraphysiologic serum E2 elevation are clinically important, since supraphysiologic serum E2 elevation may promote the tumor growth in patients scheduled for neoadjuvant chemotherapy or the recurrence of tumor in patients scheduled for adjuvant chemotherapy.

In this study, the mean age of patients was 34.7 years, and the mean AMH was 4 ng/mL. Because AMH is the first to change with age, compared to other ovarian reserve tests, it can predict changes in ovarian function most quickly [24]. The mean age of patients was younger and the mean AMH level was higher, compared to previous studies including patients diagnosed with infertility and performing ovarian stimulation for IVF procedures [22]. This can be attributed to the fact that the development of diagnostic technology has led to early diagnosis of estrogen-dependent cancers [2], and more than half of the patients in this study have implemented COS for fertility conservation rather than pregnancy purposes. As a result, the group of patients is different from previous studies, in which DOR patients account for a significant proportion [22].

Demographic studies have shown a social trend of increasing ages at marriage and delayed childbearing worldwide [25]. Conversely, as the age at which estrogen-dependent cancers are diagnosed becomes younger [2], the proportion of patients who fail to complete childbirth at the time of diagnosis will increase. Therefore, the proportion of patients with estrogen-dependent cancers undergoing COS for the purpose of preserving fertility will increase gradually.

The mean basal E2 level was 52.0 pg/mL in this study, which was higher than that in conventional IVF cycles because a number of random-start COS cycles were included in this study [22]. According to the previous studies, random start COS can start cycles regardless of the menstrual cycle without any impact on outcome compared to conventional COS, thus is useful because it minimizes delay of cancer treatment [26].

In previous studies, where 5 mg of letrozole was used with gonadotropins to prevent the elevation of E2 above physiological concentration, the mean serum E2 level on hCG triggering day was between 380–483 pg/mL [13, 17, 19]. In comparison, the mean serum E2 level on hCG triggering day in this study was 290.3 pg/mL, slightly lower than previous studies. This is believed to be due to the relatively weak ovarian response to COS, as this study included considerable cases of infertility due to DOR, unlike previous studies that performed the analysis on patients who performed COS for the purpose of preserving fertility.

Supraphysiologic level of serum E2 was found in 21.9% of cycles despite administration of letrozole 5 mg/day to reduce estradiol level. This suggests that even though 5 mg of letrozole is administered daily during COS, one fifth of all patients undergoing COS may be exposed to increased E2 above physiologic concentration, which may increase the risk of tumor growth and recurrence.

In univariate analysis, early monitoring serum E2, early monitoring number of follicles ≥ 10 mm was found to be associated with supraphysiologic elevation of serum E2 on hCG triggering day. Multiple logistic regression analysis showed that early monitoring serum E2 was an independent predictor of supraphysiologic elevation of serum E2 on hCG triggering day. Duration of stimulation, total dose of FSH and starting dose of FSH were not associated with serum E2 level on hCG triggering day. This suggests that supraphysiologic elevation of serum E2 cannot be prevented even if COS is performed with a low dose of gonadotropin for a short period of time.

When COS was performed with 5 mg of letrozole and gonadotropins, 37.5% (36/96) of the patients had a serum E2 level above cut-off value 84.5 pg/mL at the early stimulation period. Among these patients, 41.7% (15/36) actually showed supraphysiologic serum E2 level on hCG triggering day (Fig 3). Therefore, when early monitoring serum E2 level is higher than 84.5 pg/mL during COS with 5 mg of letrozole in patient with estrogen-dependent cancer, up-titration of letrozole can be considered and it might save 71.4% (15/21) of the patients from the risk of tumor growth and recurrence.

Letrozole inhibits aromatase activity specifically and maximal suppression of serum E2 concentration is achieved within two-three days [27, 28]. The half-life of letrozole is only 45 hours, and also side effects are rare and mild [29]. In multiple-dose trials, a maximum dose of 10 mg was well tolerate [30]. Since the suppression is dose-dependent, dose up-titration of letrozole up to 7.5 mg/day might be beneficial and safe in patients with early monitoring serum E2 ≥84.5 pg/mL who are at high-risk of supraphysiologic serum E2 elevation.

The limitation of this study is retrospective cohort design at a single center. Therefore, racial and regional differences should be considered in applying the results, and the results of the study should be interpreted in consideration of selection biases, etc. Because this study was conducted in the patients with estrogen-dependent cancers who performed COS for oocyte and embryo cryopreservation for the purpose of preserving fertility or pregnancy, the results of this study cannot be extended to other COS protocols such as ovarian stimulation using letrozole for the purpose of improving ovarian response [31, 32].

In conclusion, early monitoring serum E2 level is an independent predicting factor for supraphysiologic level of serum E2 on hCG triggering day in patients with estrogen-dependent cancers undergoing COS with letrozole. The cut-off level of early monitoring serum E2 was 84.5 pg/mL. To prevent supraphysiologic elevation of serum E2, up-titration of letrozole might be considered when the early monitoring serum E2 level is higher than 84.5 pg/mL during COS with letrozole and gonadotropins in patients with estrogen-dependent cancers. A well designed prospective randomized controlled multicenter study including more patients will be required in the future to determine whether the up-titration of letrozole is effective to prevent supraphysiologic levels of estrogen.

## Supporting information

S1 File (SAV)
